# Blastoid Mantle Cell Lymphoma in the Leukemic Phase: Resolving a Morphological Dilemma Through Flow Cytometry

**DOI:** 10.7759/cureus.103749

**Published:** 2026-02-16

**Authors:** Anirban Kundu, Sulagna Giri, Atoshi Basu

**Affiliations:** 1 Hematopathology, Apollo Hospitals, Kolkata, IND

**Keywords:** blastoid variant, differential diagnoses, flow cytometry, mantle cell lymphoma (mcl), pathogenesis

## Abstract

Leukemic conversion of nodal mantle cell lymphoma (MCL) is most often detected as incidental lymphocytosis on routine blood counts, often mimicking chronic lymphocytic leukemia, with flow cytometric immunophenotyping being the gold-standard test for diagnosis. Blastoid MCL closely mimics acute lymphoblastic leukemia (ALL), thus posing significant diagnostic challenges. Blastoid transformation of MCL is usually associated with *TP53 *mutations, homozygous deletions of *CDKN2A *and *CDKN2C*, amplifications and overexpression of *CDK4*,* *and occasionally microdeletions of *RB1*, and is associated with an aggressive disease course. Equivocal cases require histopathological evaluation, fluorescent in-situ hybridization, or molecular studies for confirmation. We report the case of an 82-year-old female who presented with lower respiratory tract infection and was found to have severe anemia (hemoglobin: 45 g/L) and marked leukocytosis (325 × 10⁹/L) with 63% blastoid-appearing cells on peripheral blood smear, initially suggestive of acute leukemia. Flow cytometric immunophenotyping demonstrated bright CD45 expression with low side scatter and positivity for CD19, CD20, CD38, CD5, CD79b, and FMC7 with negativity for CD34, CD23, CD200, and CD10, suggesting blastoid transformation of MCL rather than de novo ALL. This case highlights the critical role of flow cytometry in distinguishing blastoid MCL from acute leukemia, thereby preventing misdiagnosis and ensuring appropriate therapeutic decision-making.

## Introduction

Mantle cell lymphoma (MCL) is an aggressive form of B-cell non-Hodgkin lymphoma (B-NHL) arising from naïve pre-germinal center B cells and is usually characterized by small to intermediate-sized atypical lymphoid cells. The primary genetic event predominantly constitutes a translocation involving the *Cyclin D1* locus on chromosome 11q13 and the *IgH *locus on chromosome 14q32, resulting in overexpression of the Cyclin D1 protein [1]. Rarer cases may involve the *Cyclin D2* and the *Cyclin D3* loci as the driver event [2]. Leukemic conversion of nodal MCL has been described in the literature and is often detected as incidental lymphocytosis on routine blood counts. More common morphological mimickers, such as chronic lymphocytic leukemia (CLL), pose a diagnostic challenge, and flow cytometric immunophenotyping usually clinches the diagnosis. Blastoid transformation of MCL is associated with secondary genetic events, including *TP53* mutations, homozygous deletions of *CDKN2A *and *CDKN2C*, amplifications and overexpression of *CDK4*, and occasionally microdeletions of *RB1 *[3]. Blastoid variant of MCL, as the name suggests, shows atypical lymphoid cells in the peripheral blood and bone marrow, which mimic lymphoblasts and are associated with an aggressive course. Distinction from acute lymphoblastic leukemia (ALL) by flow cytometric analysis is essential for therapeutic decision-making and disease prognostication. Rare equivocal cases may warrant further molecular studies or fluorescent in-situ hybridization (FISH) for the detection of *Cyclin D1* and *IgH *translocation, along with analysis of bone marrow and lymph node tissue sections.

## Case presentation

An 82-year-old female patient presented to the Emergency Department with B-symptoms (low-grade fever and weight loss), features of lower respiratory tract infection, and no other relevant clinical history. Clinical examination revealed bilateral cervical, supraclavicular, and axillary lymphadenopathy, with no evidence of any organomegaly. As part of a routine investigation, a complete blood count was obtained (Table 1).

**Table 1:** Complete blood count (CBC) parameters along with the standard reference ranges.

CBC parameter	Patient value	Reference range [4,5]
Hemoglobin	45 g/L	Male: 135–175 g/L; female: 120–160 g/L
Total leucocyte count	325.2 × 10⁹/L	4.5–11.0 × 10⁹/L
Differential leucocyte count		
Neutrophils	02%	40–60%
Lymphocytes	96%	20–40%
Monocytes	01%	02–08%
Eosinophils	01%	00–04%
Basophils	00%	00–01%
Absolute neutrophil count	6.5 × 10⁹/L	1.5–8.0 × 10⁹/L
Absolute lymphocyte count	312.2 × 10⁹/L	1.0–4.0 × 10⁹/L
Platelet count	256 × 10⁹/L	150–400 × 10⁹/L

**Figure 1:**
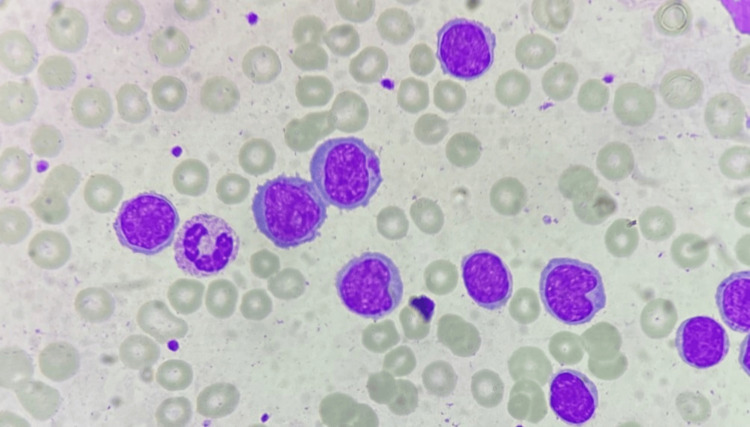
Leukocytosis with presence of large atypical lymphoid cells appearing blastoid and monocytoid in morphology. These cells have a high nuclear-to-cytoplasmic ratio, indented nuclear membrane, and slightly clumped chromatin, with few showing conspicuous nucleoli and scant to moderate pale basophilic cytoplasm (peripheral blood smear, ×1,000).

Peripheral smear examination revealed 63% atypical cells, which appeared blastoid and monocytoid in morphology, raising a suspicion for acute leukemia. The morphology of these cells is demonstrated in Figure 1.

The patient’s biochemical parameters, including liver and renal function tests, were within normal limits. Her lactate dehydrogenase levels were found to be 246 IU/L (reference range: 110-295 IU/L).

**Figure 2:**
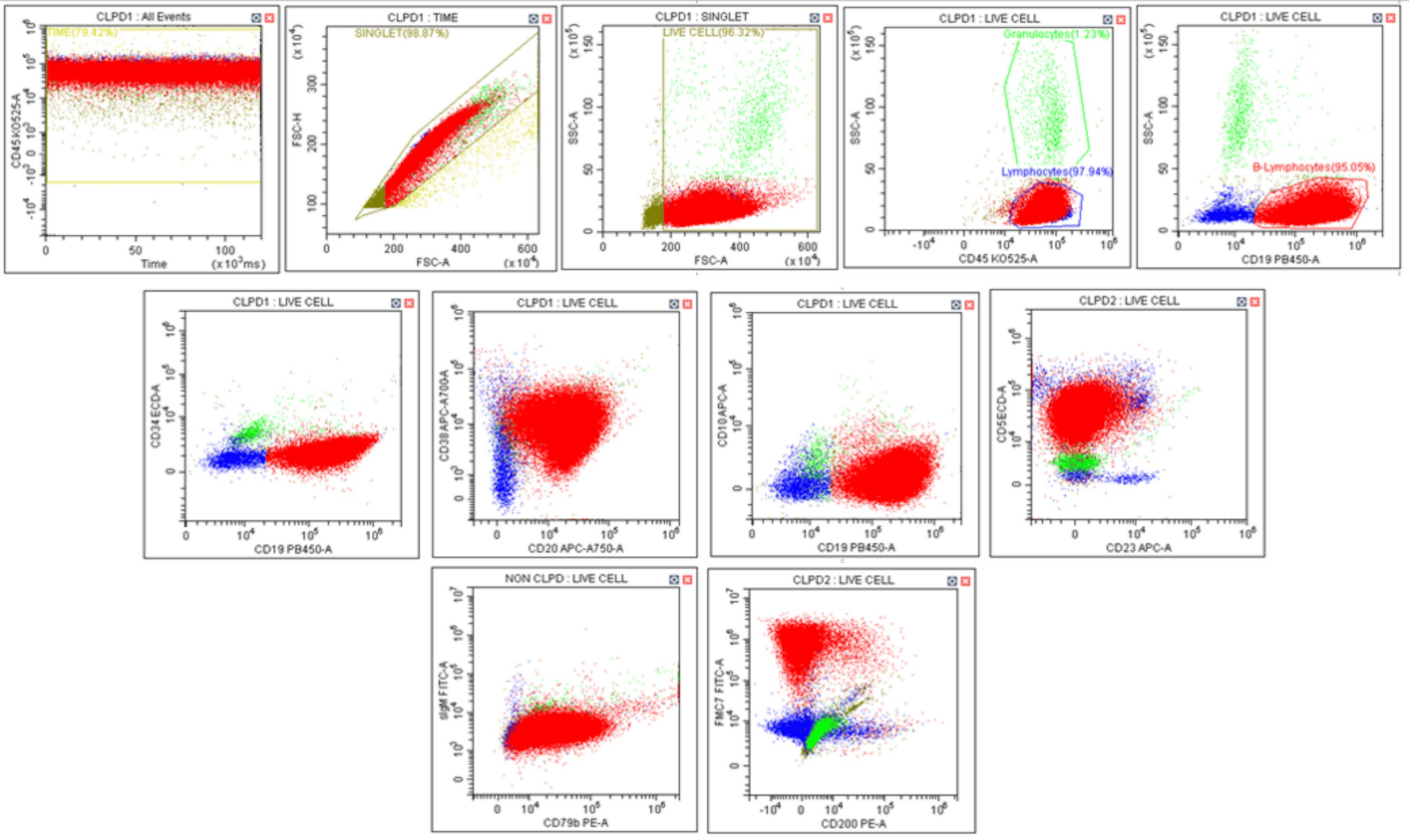
Flow cytometry revealing an abnormal lymphoid population with bright CD45 expression and low side scatter, which are brightly positive for CD19. These cells expressed CD20 (bright), CD38 (dim), CD5 (moderate), CD79b (moderate), and FMC7 (bright).

Flow cytometry was performed on the peripheral blood sample using a standard antibody panel and revealed 98% cells in the lymphocyte window with low side scatter and bright CD45 expression. An abnormal CD19 (bright)-positive population was gated in the lymphocyte gate, which further expressed CD20 (bright), CD38 (dim), CD5 (moderate), CD79b (moderate), and FMC7 (bright), along with lambda light chain restriction, as well as negativity for CD34, CD23, CD200, and CD10, thus suggesting a diagnosis of MCL. The bright CD45 expression, coupled with light chain restriction and negative CD34 expression, favored a diagnosis of blastoid MCL over ALL. The flow cytometry findings are illustrated in Figure 2.

Although molecular studies and FISH were suggested, they were not performed due to the patient’s refusal. Lymph node excisional biopsy and bone marrow studies were also not performed.

**Figure 3:**
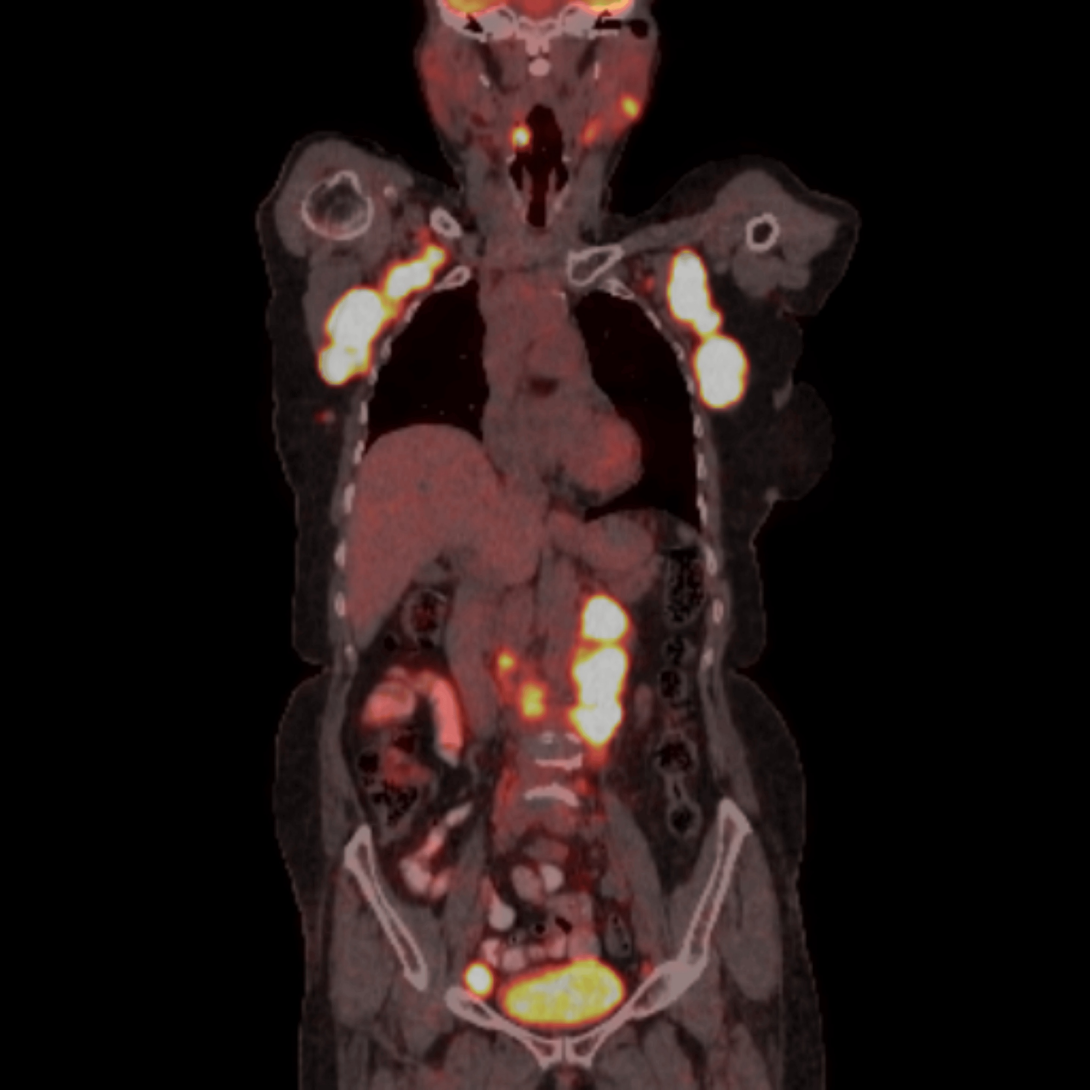
Coronal hybrid fused fluorodeoxyglucose (FDG) positron emission tomography-computed tomography (PET-CT) images revealing multiple FDG-avid supra and infra-diaphragmatic lymph nodes, suggesting involvement by high-grade non-Hodgkin lymphoma.

A fluorodeoxyglucose (FDG) positron emission tomography-computed tomography (PET-CT) scan was performed, which showed both supra-diaphragmatic and infra-diaphragmatic FDG-avid lymph nodes. The PET-CT images are in Figure 3.

The patient was started on the R-CHOP (rituximab, cyclophosphamide, doxorubicin, vincristine, and prednisone) regimen and had been planned for an autologous stem cell transplant (ASCT). However, the patient was lost to follow-up after receiving only a single cycle of R-CHOP.

## Discussion

MCL accounts for approximately 3-10% of B-NHLs and is known to arise from pre-germinal center naïve B cells. Morphologically, MCL is characterized by the presence of small to intermediate-sized atypical lymphoid cells, which commonly mimic CLL. Blastoid and pleomorphic transformation of classical nodal MCL is associated with secondary genetic events and a high proliferation rate, exhibiting frequent atypical mitoses on tissue sections and a high Ki-67 labelling index. Various morphological variants, including cells resembling lymphoblasts or having a monocytoid appearance, can be observed [6]. In concordance with existing literature, the index case also showed variable morphology with the atypical lymphoid cells exhibiting both blastoid and monocytoid morphology.

Clinical presentation usually includes B-symptoms, hepatosplenomegaly, and, more rarely, may present with acute symptoms such as respiratory distress [1]. B-cell ALL, which is the most common morphological mimicker, arises from precursor B-cell lymphoblasts and is characterized by dim CD45 expression and the presence of immaturity markers, namely, CD34 and TdT. Blastoid MCL, while having a morphology similar to L1 lymphoid blasts, still retains the more mature B-cell immunophenotype [7]. The distinction between the blastoid variant of MCL and B-ALL becomes mandatory owing to differences in disease biology, nature of disease progression, and treatment strategies. Precursor B-cell neoplasms commonly lack expression of CD20, and, as such, rituximab-based regimens are not useful. In general, B-ALL is treated by a multi-phase chemotherapeutic regimen (induction, consolidation, and maintenance), such as the Berlin-Frankfurt-Münster protocol. Blastoid MCL, on the other hand, being CD20 positive, is usually treated with a rituximab-based regimen, such as R-CHOP, while less aggressive mature B-cell neoplasms are treated with the BR (bendamustine and rituximab) regimen [6,8]. The difference in therapeutic regimen makes the distinction between a precursor neoplasm and a mature neoplasm with blastoid morphology all the more essential in a clinical context.

Diagnosis often becomes more challenging due to immunophenotypic variations associated with blastoid transformation, for instance, CD10 positivity and loss of CD5 [9]. The index case did not reveal any alterations in the immunophenotypic expression.

Another differential diagnosis of blastoid MCL includes diffuse large B-cell lymphoma, in particular the centroblastic variant. However, the characteristic immunophenotype of MCL is crucial for distinction. Also noteworthy is the fact that equivocal cases showing aberrancy in immunophenotypic expression may require demonstration of Cyclin D1 overexpression by immunohistochemistry or the demonstration of *Cyclin D1::IgH* translocation by FISH or molecular studies [10]. While tissue diagnosis remains the gold standard, the index case revealed a classical immunophenotype consistent with MCL, despite being in the blastoid phase, and did not undergo further workup despite advice from the pathologist. The immunophenotyping of MCL with its morphological mimickers is illustrated in Table 2 [11].

**Table 2:** Immunophenotypic characteristics of MCL and its mimickers. MCL = mantle cell lymphoma; B-ALL = B-cell acute lymphoblastic leukemia; CLL = chronic lymphocytic leukemia; DLBCL = diffuse large B-cell lymphoma

Immunophenotypic marker	MCL	B-ALL	CLL	DLBCL
CD45	++	-/+	++	++
CD19	++	++	++	++
CD20	++	-/+	+	++
CD5	+	-	+	-/+
CD10	-/+	+/-	-	+/-
CD23	+	-	+	-/+
CD200	-/+	-/+	++	-/+
FMC-7	+	-	-	+
CD34	-	+/-	-	-
TdT	-	+/-	-	-/+
sIg	++	-	+/-	++

Blastoid MCL is associated with an adverse prognosis and poor overall survival. Leukemic conversion and high total leucocyte counts are associated with one of the worst outcomes among all B-cell-origin NHLs. Aggressive disease course, refractoriness to standard chemotherapy, and treatment failure are among the hallmarks of the disease [1,7]. The index case had been planned for ASCT, considering the grave nature and rapid progression of the disease; however, she was lost to follow-up.

## Conclusions

Blastoid MCL is a rare and aggressive variant of MCL associated with frequent treatment failures. Variable clinical features and morphological heterogeneity are associated with difficulty in diagnosis and early recognition, in particular, due to a vast degree of morphological overlap with more common mimickers. Even though a morphological dilemma exists, immunophenotyping by flow cytometry offers a relatively simple and practical solution to arrive at a conclusive diagnosis, even enabling the clinician to avoid a more invasive procedure such as a bone marrow or lymph node biopsy. Immunophenotypic aberrations in advanced cases may pose a challenge, and in rare equivocal cases, confirmation by a bone marrow or lymph node biopsy, immunohistochemistry, molecular techniques, and FISH may be required.
